# *Dppa3* expression is critical for generation of fully reprogrammed iPS cells and maintenance of *Dlk1-Dio3* imprinting

**DOI:** 10.1038/ncomms7008

**Published:** 2015-01-23

**Authors:** Xingbo Xu, Lukasz Smorag, Toshinobu Nakamura, Tohru Kimura, Ralf Dressel, Antje Fitzner, Xiaoying Tan, Matthias Linke, Ulrich Zechner, Wolfgang Engel, D. V. Krishna Pantakani

**Affiliations:** 1 Institute of Human Genetics, University of Goettingen, Heinrich-Dueker-Weg 12, 37073 Goettingen, Germany; 2 Department of Bio-Science, Nagahama Institute of Bio-Science and Technology, Shiga 526-0829, Japan; 3 Department of Biosciences, Kitasato University School of Science, Kanagawa 252-0373, Japan; 4 Department of Cellular and Molecular Immunology, University of Goettingen, Humboldtallee 34, 37073 Goettingen, Germany; 5 Institute of Human Genetics, Johannes Gutenberg-University Mainz, Langenbeckstraße 1, 55131 Mainz, Germany

## Abstract

Reprogramming of mouse somatic cells into induced pluripotent stem cells (iPSCs) often generates partially reprogrammed iPSCs (pre-iPSCs), low-grade chimera forming iPSCs (lg-iPSCs) and fully reprogrammed, high-grade chimera production competent iPSCs (hg-iPSCs). Lg-iPSC transcriptome analysis revealed misregulated *Dlk1-Dio3* cluster gene expression and subsequently the imprinting defect at the *Dlk1-Dio3* locus. Here, we show that germ-cell marker *Dppa3* is present only in lg-iPSCs and hg-iPSCs, and that induction with exogenous *Dppa3* enhances reprogramming kinetics, generating all hg-iPSCs, similar to vitamin C (Vc). Conversely, *Dppa3*-null fibroblasts show reprogramming block at pre-iPSCs state and *Dlk1-Dio3* imprinting defect. At the molecular level, we show that Dppa3 is associated with *Dlk1-Dio3* locus and identify that Dppa3 maintains imprinting by antagonizing Dnmt3a binding. Our results further show molecular parallels between Dppa3 and Vc in *Dlk1-Dio3* imprinting maintenance and suggest that early activation of *Dppa3* is one of the cascades through which Vc facilitates the generation of fully reprogrammed iPSCs.

## Abstract (web summary)

Reprogramming of mouse somatic cells into iPSCs often generates pre-iPSCs, low-grade iPSCs that show abnormal Dlk1-Dio3 imprinting, and fully reprogrammed, high-grade iPSCs. Here, the authors show that germ-cell marker Dppa3 enhances reprogramming kinetics, critical for the maintenance of Dlk1-Dio3 imprinting and generation of fully reprogrammed iPSCs.

## Graphical abstract

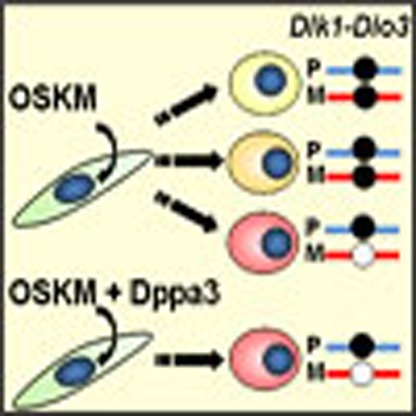

Pluripotent stem cells boast the differentiation potential virtually into any cell type of the body, and hence hold a great promise for regenerative medicine applications^1^. Recent advances in reprogramming strategies unveiled the induction of pluripotency in somatic cells by using few transcription factors resulting in the generation of induced pluripotent stem cells (iPSCs)^2^,^3^. Reprogramming of mouse somatic cells into iPSCs often generates partially reprogrammed iPSCs (pre-iPSCs), low-grade iPSCs (lg-iPSCs) that produce only low-grade chimeras and completely reprogrammed, high-grade iPSCs (hg-iPSCs) that support high-grade chimerism^4^,^5^,^6^,^7^,^8^. Pre-iPSCs are characterized by the lack of endogenous pluripotency markers expression and show residual expression of reprogramming factors, absence of chimera formation and defects at the genetic and epigenetic level^4^,^5^,^6^,^7^. Lg-iPSCs are morphologically indistinguishable from hg-iPSCs; however, they show abnormal hypermethylation of the imprinted *Dlk1-Dio3* locus and contribute to low-grade chimeras with or without germline transmission^8^,^9^. The transcriptome analysis of lg-iPSCs in comparison to embryonic stem cells (ESCs) revealed that the expression of coding and non-coding genes encoded by the *Dlk1-Dio3* imprinting cluster is misregulated due to the aberrant acquisition of DNA methylation at the maternal allele along with the normally methylated paternal allele^8^. Recently, addition of vitamin C (Vc) during reprogramming was shown to result in iPSCs with normal *Dlk1-Dio3* imprinting, yet the factor expressed in a Vc-dependent manner was not identified^10^.

Genomic imprinting is an epigenetic phenomenon established during gametogenesis and involves differential DNA methylation and post-translational histone modifications. Short DNA sequences called imprinting control regions (ICRs) are methylated on either the maternal or paternal allele to regulate expression of the imprinted gene in *cis*^11^. These marks lead to exclusive or preferential parent-specific monoallelic expression of imprinted genes^12^,^13^. During embryonic development, a pool of primordial germ cells (PGCs) gives rise to progenitors of adult gametes. After their specification and up on their arrival at the genital ridge, these PGCs undergo demethylation of the whole genome including an erasure of parent-specific methylation marks of imprinted genes, the so-called imprints^14^. The reestablishment of genomic imprinting in germ cells (GCs) according to the sex of the embryo is initiated after the entry of PGCs into gonads and continues through germ-cell differentiation^15^,^16^.

The imprint acquisition according to the sex of the embryo is regulated by several components, such as primary sequence specificity, chromatin configuration, non-histone proteins and transcriptional events (reviewed by Arnaud^17^). Specifically, the *de novo* DNA methyltransferase Dnmt3a and its related protein Dnmt3l that has no methyltransferase activity, were shown to be essential for imprint establishment at several imprinted loci^18^,^19^,^20^. In addition to the DNA methylation machinery, several other DNA-binding proteins, such as Zfp57, Nlrp2, Nlrp7, Ctcfl and Prmt7, are implicated in the establishment of imprints in a sex-specific manner^21^,^22^,^23^,^24^,^25^. Once established in GCs, several factors are known to faithfully maintain and transmit the imprints during the early stages of embryogenesis to all somatic lineages (reviewed by Arnaud^17^). *Dppa3*/*PGC7* is one such factor expressed mainly in GCs and known to protect some of the maternal as well as paternal imprints during the wave of DNA demethylation occurring in early embryogenesis^26^.

Previously, we have shown that GC marker genes, such as *Blimp1*, *Fragilis* and *Dppa3*, are expressed in all pluripotent cell types and emerge early during somatic cell reprogramming into iPSCs^27^. In the present study, we evaluate whether these GC marker genes, in particular *Dppa3*, play any role in the maintenance of *Dlk1-Dio3* imprinting during the generation of iPSCs. Interestingly, *Dppa3* is expressed only in lg-iPSCs and hg-iPSCs, but not in pre-iPSCs. Reprogramming studies in the presence of *Dppa3*, in addition to classical reprogramming factors (OSKM), show that supplementation of *Dppa3* enhances reprogramming kinetics and generates all hg-iPSCs. In line with these observations, reprogramming studies with *Dppa3*-knock out (*Dppa3*-KO) fibroblasts reveal a reprogramming arrest in pre-iPSC state along with *Dlk1-Dio3* imprinting defect. At the molecular level, we observe that Dppa3 is required for the suppression of virus-mediated reprogramming factors and endogenous retroviral elements (ERVs). Furthermore, Dppa3 is found to be associated with the intergenic differentially methylated region (IG-DMR) of the *Dlk1-Dio3* and to counteract the binding of Dnmt3a to this region during reprogramming.

## Results

### Dppa3 is present in lg-iPSCs and hg-iPSCs, not in pre-iPSCs

**Figure 1:**
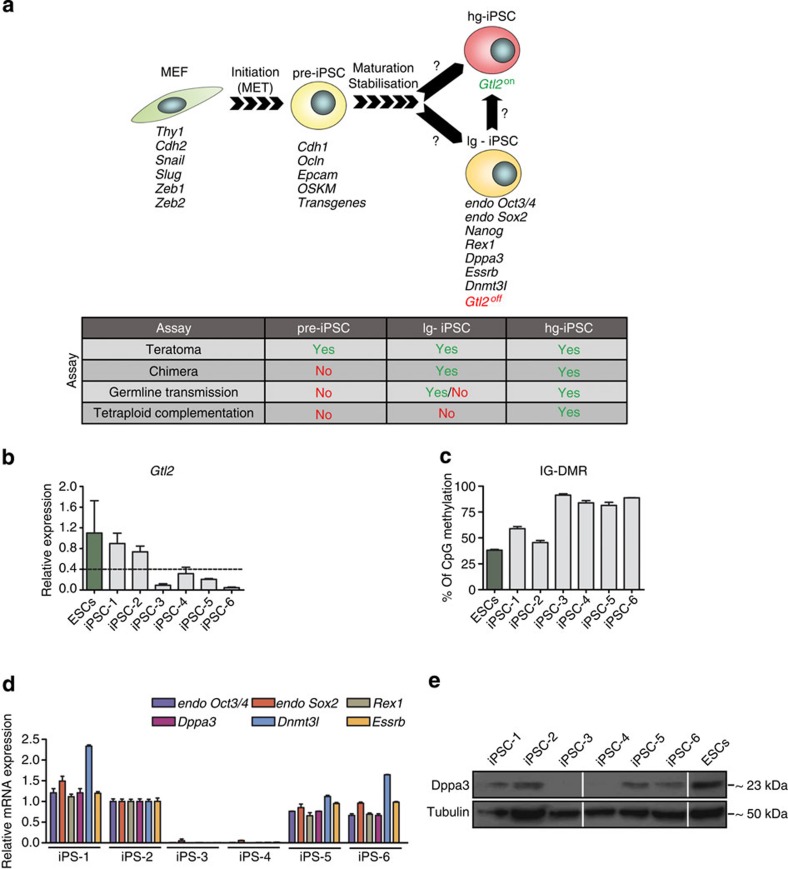
Stages of somatic cell reprogramming and Dppa3 expression status. (**a**) Schematic representation of somatic cell reprogramming, in which somatic cell (mouse embryonic fibroblast, MEF) transduced with OSKM reprogramming factors passes through initiation, maturation and stabilization phases to establish fully reprogrammed iPSC. Failure in proceeding to maturation and stabilization phases results in pre-iPSC. Similarly, defects occurring during maturation and stabilization phases result in lg-iPSC. Finally, faithful progression through all these phases results in establishment of hg-iPSC. The factor(s) responsible for conversion from pre-iPSC to either lg-iPSC or hg-iPSC, as well as from lg-iPSC to hg-iPSC are not known (?). Expression of several marker genes are indicated below each stage and pluripotency capabilities of various stages are indicated in a table. MET, mesenchymal-epithelial transition. (**b**) qRT–PCR data showing expression of *Gtl2* RNA above the threshold (dotted line) of expression typically found in ESCs, in only two iPSC clones generated with classical Yamanaka factors (OSKM). *Gtl2* RNA expression and associated error bars, representing mean±s.d. (*n*=3), were normalized to expression level in ESCs (green). (**c**) DNA methylation analysis of the *Dlk1-Dio3* IG-DMR in OSKM-derived iPSC clones showed normal methylation levels of 40–60% only in iPSC-1 and -2, whereas iPSC-3, -4, -5 and -6 displayed hypermethylation. Genomic DNA from ESCs (green) served as a control. Error bars represent mean±s.d. (*n*=2). (**d**) qRT–PCR data showing expression of various pluripotency marker genes only in iPSC-1, -2, -5 and -6, but not in iPSC-3 and -4. Gene expression and associated error bars, representing mean±s.d. (*n*=3), were normalized to expression level in iPS-2. (**e**) Western blot analysis showing expression of Dppa3 in all iPSCs, with the exception of iPSC-3 and -4. ESC protein extract was used as a control.

Advances in understanding the process of somatic cell reprogramming towards iPS cells have proposed three phases in reprogramming: *initiation*, marking mesenchymal-to-epithelial transition; *maturation and stabilization*, with pre-iPSCs and characterized by silencing of exogenous reprogramming factors and activation of endogenous pluripotency-related genes, such as *Dppa3*, *Sox2* and *Dnmt3l*^7^ (Fig. 1a). Progression through these phases generates fully reprogrammed hg-iPSCs, while failure to undergo the faithful reprogramming process yields pre-iPSCs or lg-iPSCs with imprinting defect at the *Dlk1-Dio3* locus^4^,^6^,^7^,^8^,^9^. However, the molecular mechanisms and the determining factor(s) of these cell states are yet to be identified.

To decipher the cause of aberrant *Dlk1-Dio3* imprinting, we established several iPSC lines from mouse embryonic fibroblasts (MEFs) using the classical Yamanaka’s method^3^. We found clones (iPSC-1 and -2) that displayed mRNA expression of *Gtl2* (also known as *Meg3*), a maternally expressed non-coding transcript from the imprinted *Dlk1-Dio3* locus, above the threshold level typically found in ESCs (*Gtl2*^on^)^8^, and other clones (iPSC-3, -4, -5 and -6) that showed *Gtl2* mRNA expression below that level (*Gtl2*^off^) (Fig. 1b). Consistent with the expression pattern of *Gtl2*, iPSC-1 and -2 showed normal, 40–60% DNA methylation at the *Dlk1-Dio3* IG-DMR, whereas the other clones showed DNA hypermethylation (Fig. 1c). Then we analysed the mRNA expression of pluripotency-related genes in all six iPSC lines and detected no expression in iPSC-3 and -4, but similar expression levels to control were found in iPSC-1, -2, -5 and -6 (Fig. 1d). These results led us to conclude that iPSC-1 and -2 are fully reprogrammed iPSCs, while iPSC-3 and -4 are in pre-iPSC state and iPSC-5 and -6 are in lg-iPSC state.

Recently, we showed that the expression of *Dppa3/PGC7*, a GC marker gene, precedes the expression of endogenous pluripotency marker genes, such as *Oct3/4*, *Sox2*, *Nanog* and *Klf4*, during somatic cell reprogramming^27^. *Dppa3* is known to protect some maternal as well as paternal imprints during the first wave of DNA demethylation occurring in early embryogenesis and was also implicated in the regulation of DNA methylation at ERVs, such as intracisternal A-particles (IAPs)^26^. To determine whether *Dppa3* functions in establishment/maintenance of *Dlk1-Dio3* imprinting during somatic cell reprogramming, we analysed iPSC colonies for the Dppa3 RNA and protein expression. The expression of Dppa3 was observed both in lg-iPSCs and hg-iPSCs, but not in pre-iPSCs (Fig. 1d,e). In line with these results, protein expression analysis of two *Gtl2*^off^ (R21-5-OFF and 159-3-OFF) clones and one *Gtl2*^on^ clone (R-21-4-ON) from the study of Stadtfeld *et al*.^8^, detected the expression of Dppa3 in all the three clones, indicating that the *Gtl2*^off^ clones from their study might be lg-iPSCs (Supplementary Fig. 1a). To check if *Dppa3*-deficient ESCs^26^ show any imprinting defect at the *Dlk-Dio3* locus, we performed RNA expression analysis for *Gtl2* and *Dlk1* and found no obvious differences in their expression levels between *Dppa3*-deficient and control ESCs (Supplementary Fig. 1b). Further, DNA methylation analysis of the *Dlk1-Dio3* IG-DMR revealed no significant differences with ~50% methylation levels in *Dppa3*-deficient and control ESCs (Supplementary Fig. 1c). One imprinted paternally methylated locus, *H19*, was hypermethylated in both ESC types, whereas another, *Rasgrf1*, showed 40–57% methylation in control and hypomethylation in *Dppa3*-deficient, ESCs (Supplementary Fig. 1c).

### Dppa3 enhances reprogramming and generates all hg-iPSCs

**Figure 2:**
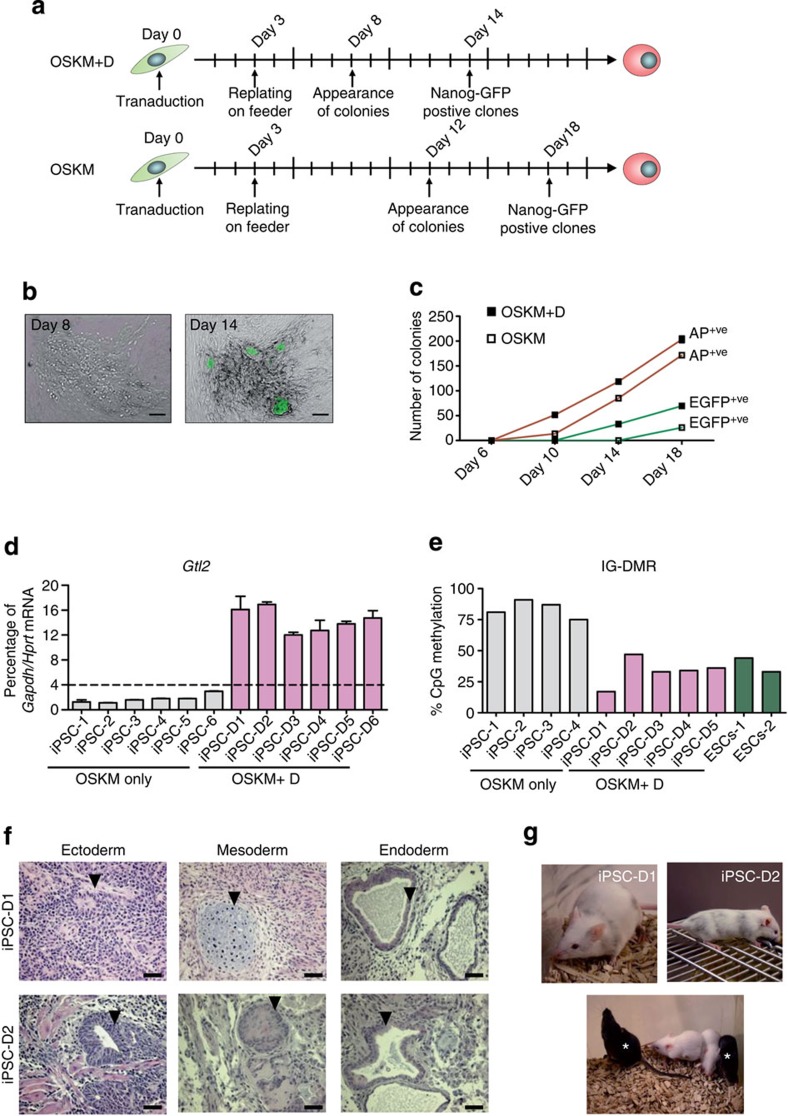
Dppa3 enhances reprogramming and generates fully pluripotent iPSCs. (**a**) Time scale showing the start of viral transduction followed by the appearance of ESC-like and *Nanog*-EGFP-positive colonies during iPSC generation using either OSKM or OSKM in combination with Dppa3 (OSKM+D). (**b**) Bright-field image showing the appearance of ESC-like colonies by day 8 of reprogramming using OSKM+D (left panel). The same colony was positive for *Nanog*-driven EGFP on day 14 (right panel). Scale bars correspond to 100 μm. (**c**) Line graph showing the number of AP-positive (AP^+ve^) and EGFP-positive (EGFP^+ve^) colonies during the reprogramming time course in the presence of OSKM or OSKM+D. (**d**) *Gtl2* RNA expression analysis in iPSCs generated using either OSKM (grey) or OSKM+D (purple). *Gtl2* RNA expression and associated error bars, representing mean±s.d. (*n*=3), were normalized to housekeeping genes, *Gapdh* and *Hprt*, and presented as percentage of expression. (**e**) DNA methylation analysis of *Dlk1-Dio3* IG-DMR in OSKM-only (grey) and OSKM+D (purple) iPSCs. ESC genomic DNA (green) was used as a control. (**f**) Light micrographs of hematoxylin and eosin-stained sections of teratomas obtained from OSKM+D iPSCs showing the presence of cell type derivatives of all three germ layers. Arrowheads indicate presence of representative tissue/cell type in respective germ layer. Scale bars correspond to 200 μm. (**g**) Images of chimeras obtained from OSKM+D iPSCs (upper panel) and their F1 progeny resulting from germline transmission (lower panel). *, pup derived from the germline-competent OSKM+D iPSCs.

To analyse the effect of exogenous *Dppa3* expression on maintenance of *Dlk1-Dio3* imprinting, we reprogrammed *Nanog*-EGFP MEFs with *Dppa3* (D) and Yamanaka factors (OSKM). OSKM+D-transduced MEFs formed colonies already by day 8 and activated *Nanog*-EGFP by day 14 of reprogramming, whereas OSKM-only-transduced cells formed colonies only by day 12 and were EGFP-positive by day 18 (Fig. 2a,b). OSKM+D transduction greatly enhanced the number of alkaline phosphatase (AP)- and EGFP-positive colonies (Fig. 2c). The *Gtl2* mRNA expression analysis in OSKM+D iPSC colonies revealed that the addition of Dppa3 results in 100% *Gtl2*^on^ colonies, while OSKM-only generated *Gtl2*^off^ colonies exclusively (Fig. 2d). The *Dlk1-Dio3* IG-DMR showed moderate hypomethylation in OSKM+D *Gtl2*^on^ and hypermethylation in OSKM-only-derived *Gtl2*^off^, iPSCs (Fig. 2e). To confirm *Dppa3*-mediated maintenance of *Dlk1-Dio3* imprinting, we generated additional OSKM+D clones. All were *Gtl2*^on^ (Supplementary Fig. 2a) and expressed paternally expressed *Dlk1* (Supplementary Fig. 2b) and several *Dlk1-Dio3* cluster-encoded miRNAs (Supplementary Fig. 2c–e) at ESC-comparable levels. We further confirmed AP, *Nanog*-EGFP and pluripotency marker genes expression in several OSKM+D clones (Supplementary Fig. 2f,g). These clones generated teratomas consisting of all three germ-layer derivatives and chimeras with germline transmission (Fig. 2f,g). Genotyping of F1 progeny confirmed the presence of OSKM+D constructs (Supplementary Fig. 3). Next, we investigated whether *Dppa3* can replace any of the reprogramming factors during iPSCs generation. MEFs supplemented with *Dppa3*, replacing any Yamanaka factor, failed to generate iPSCs (Supplementary Fig. 4), suggesting that *Dppa3* cooperates with other reprogramming factors, but cannot drive reprogramming if any are lacking.

### Modified StemPro medium generates exclusively *Gtl2* ^on^ iPSCs

**Figure 3:**
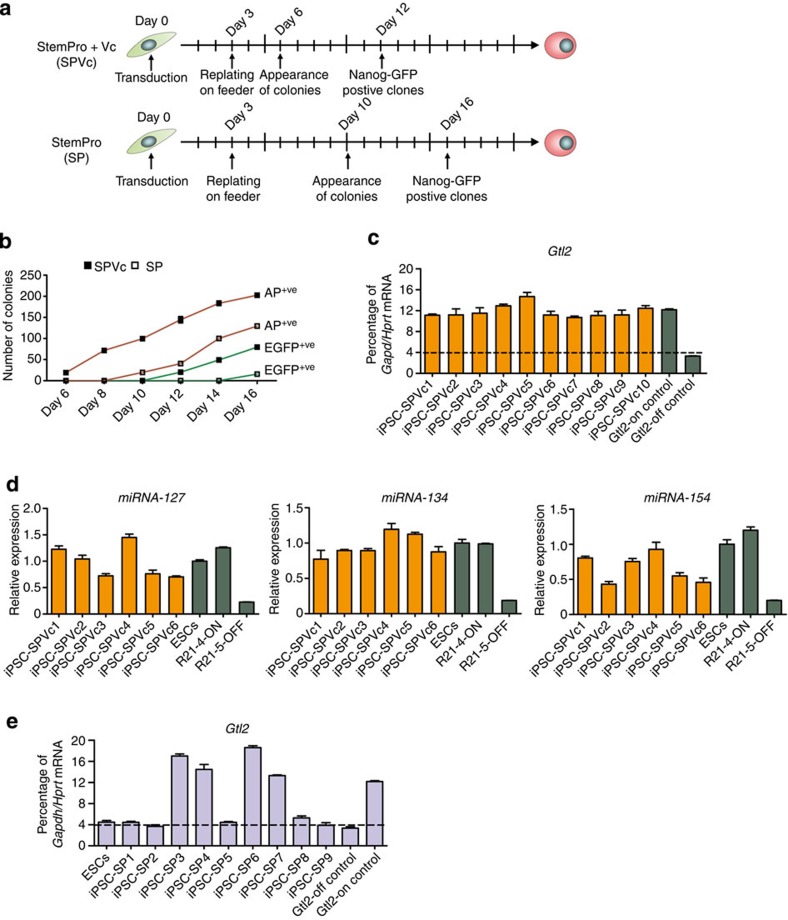
Vitamin C (Vc) enhances reprogramming and generates fully pluripotent iPSCs in low-serum medium. (**a**) Time scale showing the start of viral transduction followed by appearance of ESC-like and *Nanog*-EGFP-positive colonies during iPSC generation using OSKM in the low-serum medium StemPro (SP) in the presence (SPVc) or absence (SP) of supplementation with Vc. (**b**) Line graph showing the number of AP-positive (AP^+ve^) and EGFP-positive (EGFP^+ve^) colonies during the reprogramming time course in the presence of SP or SPVc. (**c**) qRT–PCR data showing the expression of *Gtl2* RNA in iPSC clones generated using SPVc (iPSC-SPVc). Previously generated *Gtl2*^off^ and *Gtl2*^on^ iPSC clones were used as controls. *Gtl2* RNA expression and associated error bars, representing mean±s.d. (*n*=3), were normalized to housekeeping genes, *Gapdh* and *Hprt* and presented as percentage of expression. (**d**) qRT–PCR data showing the expression of miRNA-127, miRNA-134, and miRNA-154 in iPSC-SPVc clones. ESCs, R21-4-ON (*Gtl2*^on^) and R21-5-OFF (*Gtl2*^off^) were used as controls. *miRNA* expression and associated error bars, representing mean±s.d. (*n*=3), were normalized to expression level in ESCs. (**e**) qRT–PCR data showing the expression of *Gtl2* RNA in iPSC clones generated using SP only (iPSC-SP). Previously generated *Gtl2*^off^ and *Gtl2*^on^ iPSC clones were again used as controls. *Gtl2* RNA expression and associated error bars, representing mean±s.d. (*n*=3), were normalized to housekeeping genes, *Gapdh* and *Hprt* and presented as percentage of expression.

During the course of this study, we investigated whether StemPro-based low-serum medium (SPVc), which we regularly use for the culture of spermatogonial stem cells^28^, can generate all *Gtl2*^on^ iPSCs with greater efficiency. To this end, we reprogrammed *Nanog*-EGFP MEFs with OSKM in SPVc medium and found ESC-like colonies already by day 6 of reprogramming that finally resulted in a two-fold increase in AP- and *Nanog*-EGFP-positive colonies (Fig. 3a,b). All SPVc-derived lines analysed were *Gtl2*^on^ (Fig. 3c) and expressed maternally expressed miRNAs, validating activation of *Dlk1-Dio3* locus genes (Fig. 3d). These results led us to hypothesize that the components of SPVc help in proper maintenance of imprinting at the *Dlk1-Dio3* locus during reprogramming. The SPVc medium inherently contains Vc that was reported to protect against the loss of *Dlk1-Dio3* imprinting during reprogramming^10^. It was shown that Vc functions primarily by preserving active chromatin structure at the *Dlk1-Dio3* locus and by preventing recruitment of Dnmt3a, a *de novo* DNA methyltransferase, to this locus. Hence, we asked whether StemPro-based low-serum medium depleted of Vc (SP) can also generate exclusively *Gtl2*^on^ iPSCs with reprogramming kinetics similar to SPVc. However, the use of SP medium resulted in slower reprogramming kinetics and only four of nine iPSC clones (SP-iPSC) were *Gtl2*^on^ (Fig. 3b,e).

### *Dppa3*-deficient fibroblasts arrest in pre-iPSC state

**Figure 4:**
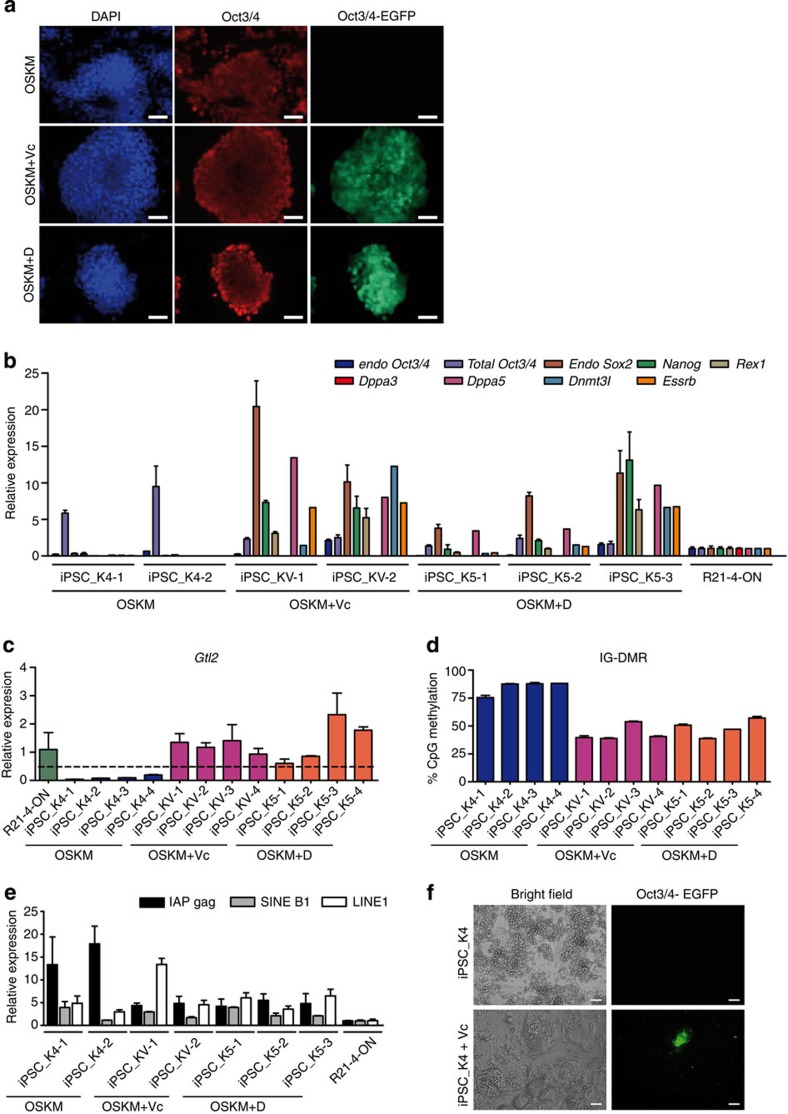
*Dppa3*-knockout (KO) fibroblasts are arrested in pre-iPSC state during reprogramming. (**a**) Immunofluorescence images showing Oct3/4-positivity and activation of *Oct3/4*-driven EGFP in *Dppa3*-KO fibroblasts reprogrammed either with OSKM (upper panel), OSKM+Vc (middle panel) or OSKM+Dppa3 (lower panel). Nuclei were counterstained with DAPI. Scale bars correspond to 50 μm. (**b**) qRT–PCR data showing the expression of various pluripotency marker genes in the indicated *Dppa3*-KO-derived iPSCs generated with OSKM+Vc and OSKM+D, but not in OSKM-only-generated iPSCs. Gene expression and associated error bars, representing mean±s.d. (*n*=3), were normalized to expression level in *Gtl2*^on^ iPSC clone^6^ (R21-4-ON). (**c**) qRT–PCR data showing expression of *Gtl2* RNA in *Dppa3*-KO-derived iPSCs in presence of OSKM (blue), OSKM+Vc (purple) or OSKM+Dppa3 (red). *Gtl2* RNA expression and associated error bars, representing mean±s.d. (*n*=3), were normalized to expression level in *Gtl2*^on^ iPSC clone^6^ (R21-4-ON). (**d**) DNA methylation analysis of the *Gtl2* IG-DMR in iPSCs generated from *Dppa3*-KO fibroblasts with OSKM-only (blue), OSKM+Vc (purple) and OSKM+Dppa3 (red). Error bars represent mean±s.d. (*n*=2). (**e**) qRT–PCR data showing expression of retrotransposons, intracisternal A-particles (IAP gag, black), short interspersed nuclear elements (SINE B1, grey), and long interspersed nuclear elements (LINE 1, white), in *Dppa3*-KO-derived iPSCs in presence of OSKM, OSKM+Vc or OSKM+Dppa3. Retrotransposons expression and associated error bars, representing mean ±s.d. (*n*=3), were normalized to expression level in *Gtl2*^on^ iPSC clone^6^ (R21-4-ON). (**f**) Bright-field image (left panel) showing colony morphology and fluorescence image (right panel) showing the activation of *Oct3/4*-driven EGFP in iPSCs derived from *Dppa3*-KO fibroblasts with OSKM (upper panel) and the same clones treated with Vc (iPSC_K4+Vc) (lower panel). Scale bars correspond to 200 μm.

On the basis of parallels between Dppa3 and Vc in preventing *Dlk1-Dio3* imprinting loss, we investigated whether Vc treatment compensates for the Dppa3 absence during reprogramming. Towards this end, we reprogrammed MEFs derived from the *Dppa3*-knockout (KO)/*Oct4*-EGFP animals (KO_MEFs) (Supplementary Fig. 5) in the presence and absence of Vc and in the presence of exogenous *Dppa3*. Reprogramming of KO_MEFs with OSKM-only resulted in iPSC-like colonies that, however, failed to activate the *Oct3/4*-driven EGFP, whereas cells reprogrammed with OSKM+Vc or OSKM+D showed efficient activation of the *Oct3/4*-EGFP (Fig. 4a). The mRNA expression analysis for pluripotency marker genes revealed that KO_MEFs reprogrammed with OSKM-only (iPSC_K4) are arrested in the pre-iPSC state, as they could not activate the endogenous pluripotency network, whereas the cells reprogrammed with OSKM+Vc (iPSC_KV) or OSKM+D (iPSC_K5) showed expression levels comparable to control cells (Fig. 4b). Silencing of exogenous reprogramming factors is crucial for proper transition from maturation-to-stabilization phase during the reprogramming and transition failure results in loss of endogenous pluripotency network activation^6^. Interestingly, OSKM-only-reprogrammed cells failed to inactivate viral transgenes, as indicated by higher levels of total *Oct3/4* mRNA levels derived from the transgene (Fig. 4b). iPSC_K4 clones showed very low *Gtl2* mRNA levels and IG-DMR hypermethylation, whereas clones derived from OSKM+Vc (iPSC_KV) or OSKM+D (iPSC_K5) conditions showed normal *Gtl2* expression and methylation (Fig. 4c,d). The DNA methylation analysis of two additional imprinted loci, *H19* and *Snrpn*, showed normal DNA methylation levels in iPSC clones form all three conditions (Supplementary Fig. 6a,b), indicating that Dppa3 depletion specifically affects the *Dlk1-Dio3* imprinting. It is known that Dppa3 suppresses ERVs such as IAPs and LINEs during early embryogenesis^26^. To determine whether ERVs are suppressed in iPSCs derived from *Dppa3*-KO fibroblasts, we performed quantitative reverse transcription PCR (qRT–PCR) analysis for their expression and found that IAPs are significantly activated in OSKM-only, but not in OSKM+Vc or OSKM+D, iPSCs (Fig. 4e).

To verify whether Vc functions in conversion of pre-iPSCs derived from KO_MEFs towards lg-iPSCs or hg-iPSCs, we cultured these cells with Vc and could observe the activation of *Oct3/4*-EGFP (Fig. 4f). These colonies resembled lg-iPSCs and showed no *Gtl2* expression at a global level (data not shown), suggesting that Vc can overcome the reprogramming block, but cannot restore the imprinting defect in the resulting lg-iPSCs. To determine whether exogenous Dppa3 can rescue the reprogramming block seen in KO_MEFs-derived pre-iPSCs, we supplemented these clones with retroviral Dppa3 and observed neither *Gtl2* activation nor morphological changes (Supplementary Fig. 7a–c).

### Vitamin C (Vc) mediates early activation of Dppa3

**Figure 5:**
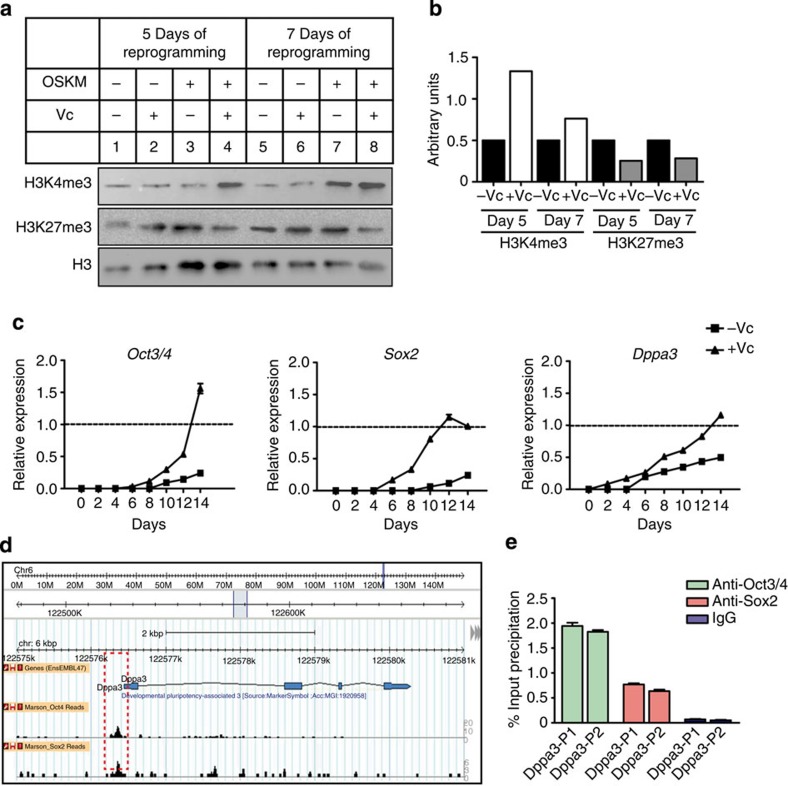
Vitamin C mediates global chromatin relaxation and early activation of pluripotency-related genes. (**a**) Western blots showing expression of histone modifications during OSKM reprogramming on days 5 and 7 in standard ESC culture medium in the presence and absence of Vc. MEFs supplemented with no OSKM or Vc (lanes 1 and 5), either Vc only (lanes 2 and 6), or OSKM only (lanes 3 and 7) served as controls. All blots were reprobed with an anti-total Histone 3 (H3) antibody, and one representative blot shows loading control. (**b**) Quantification of densitometric intensity of H3K4me3 and H3K27me3 bands shown in (**a**). (**c**) qRT–PCR data showing the expression of *Oct3/4* (left panel), *Sox2* (middle panel), and *Dppa3* (right panel) mRNAs during the OSKM reprogramming time course in standard ESC culture medium in the presence (+Vc) or absence (−Vc) of Vc. Gene expression and associated error bars, representing mean ±s.d. (*n*=3), were normalized to expression level in ESCs (dotted line). (**d**) Publicly available ChIP-Seq data showing the binding of Oct3/4 and Sox2 (black peaks) in the *Dppa3* promoter region (red box). (**e**) ChIP–qPCR analysis showing the binding of Oct3/4 (green) and Sox2 (maroon) at the *Dppa3* promoter region (amplicons Dppa3-P1 and Dppa3-P2) in ESCs. ChIP with IgG (blue) served as a negative control. The ChIP data, representing mean±s.d. (*n*=2), is presented as percent of input DNA. a.u., arbitrary units.

We hypothesized that Vc might activate *Dppa3*, which is ultimately responsible for maintenance of proper imprinting during early stages of reprogramming. To verify this hypothesis, we reprogrammed *Nanog*-MEFs using OSKM in standard ESC medium in the presence or absence of Vc and investigated changes in chromatin modifications and pluripotency marker expression. After day 5 of reprogramming, MEFs treated with Vc (+Vc) showed enrichment for H3K4me3, a histone modification mark associated with active gene transcription, whereas MEFs treated without Vc (−Vc) showed basal H3K4me3 levels (Fig. 5a,b). In contrast to H3K4me3, the levels of H3K27me3, a histone mark associated with transcription repression, were reduced in +Vc, but not in −Vc cells (Fig. 5a,b). These changes were also evident at day 7 of reprogramming (Fig. 5a,b). Endogenous *Oct3/4* and *Sox2* mRNA expression was activated at least two days earlier in +Vc than in −Vc cells (Fig. 5c). Further, by day 12 of reprogramming, we observed much higher levels of endogenous *Oct3/4* and *Sox2* mRNA levels in +Vc cells compared with −Vc cells (Fig. 5c). Surprisingly, *Dppa3* mRNA expression was already detected by day 2 of reprogramming in +Vc treated MEFs, but only by day 6 of reprogramming in control −Vc cells (Fig. 5c). Correlating early *Dppa3* activation with potential Vc-induced chromatin changes, we hypothesized that exogenous reprogramming factor(s) might gain access to the *Dppa3* promoter and activate its expression early in Vc-treated cells. To ascertain this assumption, we searched publicly available chromatin immunoprecipitation (ChIP)-Seq data for *Oct3/4*- and *Sox2*-binding sites/targets across the ESC genome (http://promotion.molgen.mpg.de/gb2/gbrowse/Mm.enhancer/) and found *Oct3/4* and *Sox2* binding sites at the *Dppa3* promoter region^29^ (Fig. 5d). To validate these global ChIP-Seq data, we performed ChIP assays with anti-Oct3/4 and anti-Sox2 antibodies and quantitative PCR (qPCR) analysis of two PCR amplicons in the *Dppa3* promoter region. The qPCR data clearly showed that Oct3/4 and Sox2 bind *Dppa3* promoter region (Fig. 5e).

### Dppa3 binds to the IG-DMR of the *Dlk1-Dio3* cluster

**Figure 6:**
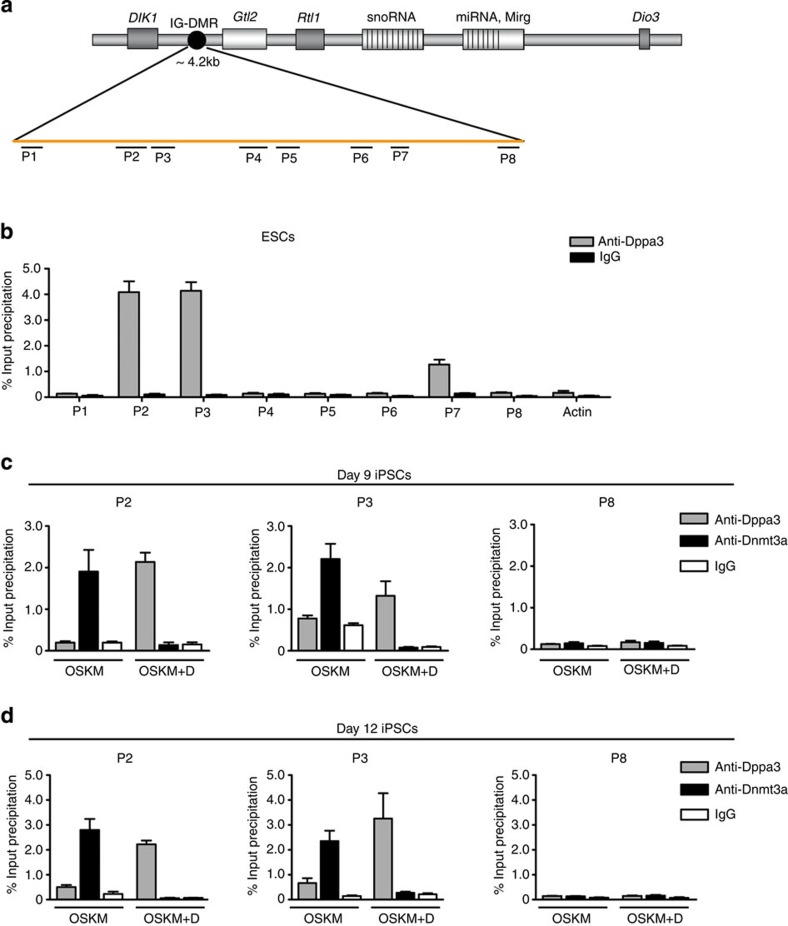
Dppa3 binds to a specific region within the IG-DMR of the *Dlk1-Dio3* imprinted cluster and prevents the recruitment of Dnmt3a. (**a**) Simplified schematic diagram showing the *Dlk1-Dio3* imprinted cluster together with paternally expressed genes (dark grey boxes) and maternally expressed genes (light grey boxes). The ~4.2 kb long IG-DMR (black circle), representing the imprinted control region, and the eight ChIP–qPCR amplicons (P1–P8) spanning the entire IG-DMR are indicated. (**b**) ChIP–qPCR analysis of Dppa3-binding sites (grey) across the eight PCR amplicons of the IG-DMR in ESCs. ChIP with IgG (black) was used as a negative control. The ChIP data, representing mean±s.d. (*n*=2), is presented as percent of input DNA. (**c**,**d**) ChIP–qPCR of Dppa3 (grey) and Dnmt3a (black) binding at the P2, P3 and P8 amplicons of the IG-DMR in OSKM and OSKM+D on day 9 (**c**) and day 12 (**d**) of reprogramming. ChIP with IgG (white) was used as a negative control. The ChIP data, representing mean±s.d. (*n*=3), is presented as percent of input DNA.

Dppa3 is considered as a DNA-binding protein and indeed *in vitro* studies have shown that it binds DNA non-specifically^26^. To test the hypothesis that Dppa3 is physically associated with the IG-DMR of the *Dlk1-Dio3* region, we performed ChIP assay with Dppa3 antibody and ESC chromatin. As the IG-DMR spans around 4.2 kb in the *Dlk1-Dio3* cluster, we designed eight PCR amplicons (P1–P8) covering the entire region (Fig. 6a). ChIP assays revealed that Dppa3 specifically binds the IG-DMR region represented by amplicons P2 and P3 (Fig. 6b). Recent data showed that Dnmt3a is highly enriched at the IG-DMR during somatic cell reprogramming; probably leading to the silencing of maternal allele and that this effect can be counteracted by the addition of Vc^10^. This led us to analyse whether exogenous Dppa3 can also counteract Dnmt3a binding at the IG-DMR. Interestingly, Dnmt3a association with P2 and P3 was significantly reduced in OSKM+D-transduced cells on day 9 of reprogramming, whereas OSKM-only-transduced cells showed Dnmt3a enrichment (Fig. 6c). Similar results were obtained at day 12 of reprogramming (Fig. 6d), confirming that Dppa3 prevents IG-DMR hypermethylation by preventing Dnmt3a binding. These results indicate that Vc facilitates *Dppa3* transcription during early stages of reprogramming leading to the initiation of maturation process, binding of Dppa3 to the *Dlk1-Dio3* cluster and restriction of Dnmt3a access to this locus.

## Discussion

By identifying Dppa3 expression in lg-iPSCs (*Gtl2*^off^) and hg-iPSCs (*Gtl2*^on^), but not in pre-iPSCs, we hypothesized that the temporal expression of Dppa3 is crucial for the maintenance of *Dlk1-Dio3* imprinting in lg-iPSCs. This assumption was supported by our observation that *Dppa3*-KO fibroblasts undergoing reprogramming were arrested in pre-iPSCs state and that the addition of Vc to these pre-iPSCs can only convert them to lg-iPSCs, which show abnormal *Dlk1-Dio3* imprinting. Further, we identified that in the absence of Dppa3, the ERVs as well as exogenous retrovirus-mediated reprogramming factors remain unsilenced leading to maturation-to-stabilization transition failure. Collectively, our results reveal that the temporal expression of Dppa3 is crucial not only for maintaining the *Dlk1-Dio3* imprinting, but also for transition from pre-iPSC-to-lg-iPSC and to establish the authentic induced pluripotency.

*Dppa3* is highly expressed during embryonic development, as well as in germ cells and pluripotent stem cells^30^,^31^. Gene ablation studies of *Dppa3* in mice revealed that it functions during early embryonic development to protect the maternal genome against the first wave of active DNA demethylation^26^. Moreover, *Dppa3* was also shown to be essential for protecting several but not all imprinted loci and ERVs against DNA demethylation during post-fertilization events^26^. Interestingly, the paternally methylated *Dlk1-Dio3* region was not affected in *Dppa3*-null fertilized oocytes, whereas *H19* and *Rasgrf1*, the two other paternally methylated imprinted loci, showed partial loss of imprints^26^. Similarly, our current analysis of *Dppa3*-deficient ESCs also revealed no obvious defects in *Dlk1-Dio3* imprinting. In contrast to these observations, *Dppa3*-deficient fibroblasts failed to reprogram, arrested in pre-iPSCs state and showed imprinting defect at the *Dlk1-Dio3* cluster. This discrepancy could be mainly due to artificial reprogramming of somatic cells versus normal early embryonic development. Another explanation would be that the oocyte-delivered Dppa3 might maintain stable *Dlk1-Dio3* imprinting in *Dppa3*^−/−^ embryos and ESCs derived thereof. Supporting this view, imprinting of paternally methylated *H19* and *Rasgrf1* is markedly disturbed in ESCs derived from *Dppa3*^−/−^ embryos, whereas the *Dlk1-Dio3* imprinting is stable and normal.

A recent report described that Dppa3 binds to nucleosomes containing an H3K9me2^32^. Our ChIP analysis showed that Dppa3 binds to a specific region in the *Dlk1-Dio3* IG-DMR, which also suggests that the interaction might involve modified histones. Unlike many imprinted loci, the IG-DMR of the *Dlk1-Dio3* cluster was reported to contain negligible H3K9me2 (ref. 32). Thus, it is possible that the association of Dppa3 with the IG-DMR is mediated by histone modifications other than H3K9me2 or another interaction protein.

Recently, Stadtfeld *et al*.^10^ showed that Vc can prevent the *Dlk1-Dio3* imprinting defect during reprogramming mainly by preserving the active chromatin structure at this locus. The resulting active chromatin was suggested to counteract the recruitment of Dnmt3a, a *de novo* DNA methyltransferase, to this locus thus maintaining the normal imprinting in Vc-treated cells^10^. Our results are in consistent with observations of Stadtfeld *et al*.^10^, indicating that Vc enhances reprogramming kinetics and prevents *Dlk1-Dio3* imprinting loss. Moreover, the comparison of reprogramming kinetics between Vc and OSKM+D showed that Vc treatment resulted in appearance of iPSC colonies at least 2 days earlier than with OSKM+D, indicating that Vc broadly impacts reprogramming. Recently, it was reported also that culturing of pre-iPSCs in the presence of Vc establishes chimera forming iPSCs; however, germline transmission and *Dlk1-Dio3* imprinting status have not been tested^4^. We observed that although Vc can overcome the pre-iPSCs arrest of Dppa3-KO cells and result in lg-iPSCs establishment, it could not establish the normal imprinting at *Dlk1-Dio3* locus. These results highlight that Vc-mediated chromatin changes or gene expression during early stages of reprogramming are crucial for maintenance of *Dlk1-Dio3* imprinting.

Dppa3’s role in preventing the *Dlk1-Dio3* imprinting defect suggests that Vc functions through *Dppa3* activation, or Vc and *Dppa3* function independently in a similar mechanistic pathway. The former cooperative model is supported by the observation that *Dppa3* transcripts are upregulated in Vc-treated cells. The acceleration and efficiency of reprogramming seen with Vc^4^,^10^,^33^ is also observed with exogenous Dppa3, indicating that Dppa3 either alone or in cooperation with other yet unknown factor(s) might implement Vc-mediated maintenance of *Dlk1-Dio3* imprinting. Moreover, the early Dppa3 expression is crucial for timely suppression of virus-mediated reprogramming factors and transition of pre-iPSC into lg-iPSC state. On the basis of our observations, we propose that early Dppa3 expression, by exogenous induction or Vc exposure, is followed by Dppa3 binding to the IG-DMR and probably to other genomic regions. Bound Dppa3 prevents Dnmt3a recruitment to the IG-DMR, maintaining the imprinting and expression of imprinted *Dlk1-Dio3* genes. In line with this hypothesis, it was recently shown that the forced expression of Dppa3 in NIH3T3 cells causes the global DNA demethylation by counteracting the recruitment of Dnmt1, a maintenance DNA methyltransferase^34^. The observed counteracting mechanism was partially mediated by Dppa3 competing for the interaction with Uhrf1/Np95, a protein known to recruit Dnmt1 to hemimethylated DNA and to maintain the DNA methylation during replication^34^,^35^,^36^. Considering these above observations along with the fact that *de novo* DNA methylation process requires cooperation between Dnmt3a and Dnmt1 enzymes^37^, we suggest that Dppa3 is functioning in a similar way to block the accession of Dnmt3a to *Dlk1-Dio3* locus during somatic reprogramming. In the second scenario, the function of Dppa3 is compensated by other Dppa family members or unknown factors, which also implement Vc-mediated imprinting maintenance.

## Methods

### Cell culture

Mouse ESCs and induced pluripotent stem cells (iPSCs) were cultured in Dulbecco’s Modified Eagle Medium (DMEM) (PAN, Germany) supplemented with 15% foetal calf serum (FCS; PAN), 2 mM L-glutamine (PAN), 50 μM β-mercaptoethanol (Gibco/Life Technologies), 1% non-essential amino acids (NEAA) (Gibco/Life Technologies), 1% sodium pyruvate (Gibco/Life Technologies), 1% penicillin/streptomycin (PAN), and 1,000 U ml^−1^ leukemia inhibitory factor (LIF) (Millipore, Germany). For reprogramming studies with low-serum medium, cells were cultured in StemPro medium (Invitrogen/Life Technologies) supplemented with StemPro supplement (Invitrogen/Life Technologies), 1% FCS, 2 mM L-Glutamine, 1 mM sodium pyruvate, 1% NEAA, 100 μM β-mercaptoethanol and 1000 U ml^−1^ LIF in the presence or absence of 50 μg ml^−1^ vitamin C (Sigma-Aldrich).

### Preparation of *Dppa3*-KO/*Oct4*-EGFP fibroblasts (MEFs)

*Dppa3*-KO mice were generated using the conventional knockout strategy, and were maintained in a 129/Sv and C57BL/6 mixed background^26^. *Oct4*-EGFP transgenic mice were maintained in a DBA2 and C57BL/6 mixed background^38^. The animals were cared for in accordance with the guidelines of Osaka University Animal Care and Use Committee. To prepare *Dppa3*-KO/*Oct4*-EGFP MEFs, *Dppa3*^−/−^ male mice carrying the *Oct4*-EGFP transgene were crossed with *Dppa3*^+/−^ female mice carrying the *Oct4*-EGFP transgene. The morning on which a copulation plug was noted was defined as embryonic day 0.5 (E0.5). MEFs were isolated from E13.5 embryos. After the removal of the head, visceral tissues and gonads, the remaining bodies were washed with phosphate-buffered saline, minced and dissociated with 0.1% Trypsin/1mM EDTA solution. MEFs from each embryo were plated on individual 0.1% gelatin-coated 100 mm dishes and incubated at 37 °C with 5% CO_2_. We used the MEFs within two passages from initial plating to avoid replicative senescence. The embryo heads were used for genotyping *Dppa3* as described previously^26^. The sex of the embyros and the presence of the *Oct4*-EGFP transgene were determined by morphology and EGFP fluorescence of gonads, respectively. *Dppa3*-KO MEFs preparations were approved by Osaka University Animal Care and Use Committee.

### Generation of iPSCs

We used retroviral expression vectors for *Oct3/4*, *Sox2*, *Klf4* and *c-Myc* either in presence or absence of *Dppa3* (ref. 3) to reprogram MEFs into iPSCs. Briefly, MEFs isolated from transgenic *Nanog*-EGFP mice^39^ or *Dppa3*-KO/*Oct4*-EGFP mice were transduced with retroviral particles as described^27^. To establish iPSC lines, colonies appearing 8–12 days after transduction were selected and cultured in 24-well plates under standard ESC culture conditions and monitored for ESC-like morphology using an Olympus IX71 inverted microscope (Olympus). Further, several independent iPSC lines were established from each combination to examine the expression of *Gtl2*, as well as for further characterization. Alternatively, iPSCs were generated in low-serum medium, as described above.

### Immunostaining and alkaline phosphatase (AP) staining

Immunostaining was performed as described previously^40^ using mouse monoclonal antibodies to SSEA1. Cytochemical staining for AP was performed using the Leukocyte Alkaline Phosphatase Kit (Sigma-Aldrich).

### Total protein extraction and western blotting

Total protein extracts were prepared using lysis buffer (10 mM Tris-HCl, pH 8.0, 1 mM EDTA, 2.5% SDS, 100 mM phenylmethylsulfonyl fluoride (PMSF)) containing protease inhibitor cocktail (Roche). Protein samples were resolved on 4–12% SDS–PAGE gels (Life Technologies) and transferred onto nitrocellulose membranes (Amersham Biosciences/GE Healthcare). Membranes were processed using standard western blot protocols, and signals were detected using a chemiluminescence kit (Santa Cruz Biotechnology). Antibody sources are listed in Supplementary Table 1.

### RNA extraction and real-time PCR (qRT–PCR) analysis

Total RNA was extracted from cells using NucleoSpin miRNA kit (Machery-Nagel) following the manufacturer’s protocols. For the mRNA quantification experiment, 5 μg total RNA was converted into complementary DNA (cDNA) using the SuperScript II System (Invitrogen/Life Technologies). For the miRNA quantification experiment, 1 μg total RNA was used for cDNA synthesis using the miScript II RT Kit (Qiagen). For qRT–PCR analysis, diluted cDNA (1/10) was used as a template in QuantiFast SYBR Green (Qiagen) assays on an ABI7900HT Real-Time PCR System (Applied Biosystems). The qRT–PCR data were normalized to the expression of housekeeping genes (*Hprt* and *Gapdh*) and presented as percent expression of the mean of the two housekeeping genes. Primers used in qRT–PCR are listed in Supplementary Table 2.

### Bisulfite sequence analysis

WT or *Dppa3*-KO ES cells were bisulfite-treated using the EpiTect Bisulfite Kit (Qiagen). To amplify the DMRs of *H19*, *Dlk1/Gtl2* and *Rasgrf1*, fully or seminested PCR was performed. The first and second rounds of PCR were performed using AccuPrime Taq DNA polymerase (Invitrogen) and ExTaq HS (Takara Bio), respectively. The first round of PCR consisted of the following cycling conditions: 2 min at 94 °C for 1 cycle and 30 s at 94 °C, 30 s at 50 °C and 1 min at 68 °C for 30 cycles. The second round of PCR consisted of the following cycling conditions: 2 min at 94 °C for 1 cycle and 30 s at 94 °C, 30 s at 55 °C and 1 min at 72 °C for 30 cycles. The sequences of the PCR primers have been described previously^26^. The PCR products were purified using the QIAEX II Gel Extraction Kit (QIAGEN), cloned into the pGEM-T Vector (Promega) and sequenced using an ABI PRISM 3100 Genetic Analyzer (Applied Biosystems).

### Methylation analysis of the *Gtl2* IG-DMR

Genomic DNA isolation from cells and bisulfite pyrosequencing were performed as previously described^41^. Briefly, Genomic DNA of MEFs, iPSCs and ESCs was extracted with the GentraPuregeneKit (Qiagen) according to the manufacturer’s instructions and quantified using a NanoDrop 2000 spectrophotometer. Bisulfite conversion of 500 ng genomic DNA per sample was performed with the EpiTect Bisulfite Kit (Qiagen) according to the manufacturer’s specifications. Quantification of DNA methylation was carried out by PCR of Bisulfite-converted DNA and pyrosequencing. PCR and sequencing primers for bisulfite pyrosequencing were designed using the Pyrosequencing Assay Design Software 2.0 (Qiagen) and are listed in Supplementary Table 2. For pyrosequencing, a PyroMark Q96 ID instrument (Qiagen) and PyroMark Gold Q96 reagents (Qiagen) were used. Data were analysed using the PyroMark CpG Software 1.0.11 (Qiagen).

### Chromatin immunoprecipitation (ChIP) assay

ChIP assays were performed on ESCs and MEFs undergoing reprogramming using the Diagenode OneDay ChIP kit (Diagenode). Briefly, the cells (3 × 10^6^ cells per pull-down) were crosslinked using formaldehyde and lysed using the shearing kit (Diagenode), followed by sonication with a bioruptor (Diagenode, UCD-200 TM) to obtain an average chromatin size of 400 bp. Then, the sheared chromatin was immunoprecipitated with 5 μg of indicated antibodies, and the Diagenode OneDay ChIP protocol was followed to extract the chromatin bound to each specific antibody. Rabbit IgG was used as a control in mock ChIP experiments. Quantitative analysis was performed using qRT–PCR and the primers listed in Supplementary Table 2. The ChIP–qPCR data were analysed using ΔCt method in which the immunoprecipitated (IPed) sample Ct value was normalized to the input DNA Ct value and the percentage of precipitation was calculated using the following formula- %input=2^-(Ct Input—Ct IPed) × dilution factor of Input DNA × 100%. All fold enrichment values are means of three independent experiments.

### Teratoma formation assay

The teratoma formation assay was performed as previously described^42^,^43^. Briefly, iPSCs (1 × 10^6^ cells) were injected subcutaneously into RAG2^−/−^γc^−/−^ mice lacking T, B and natural killer (NK) cells. Tumour growth was monitored weekly by palpation and size was measured using linear calipers. Animals were sacrificed when a tumour diameter of 1 cm was reached. Autopsies were performed and tumour tissue was placed in phosphate-buffered 4% formalin for 16 h and then embedded in paraffin. For histological analysis, the specimens were stained with hematoxylin and eosin (HE).

### Replicates

Unless otherwise stated, all results presented are representative of two or more independent experiments. All qRT–PCR data for RNA expression analysis were calculated using either the standard curve method or comparative Ct method.

## Author contributions

X.X., L.S., W.E. and D.V.K.P. conceived the experiments and analysed the data. X.X., L.S. and X.T. performed reprogramming studies and analysed the data. T.N. performed DNA methylation and qRT–PCR analyses of *Dppa3*^−/−^ ESCs. T.N. and T.K. provided *Dppa3*-KO fibroblasts. A.F., M.L. and U.Z. performed DNA methylation analysis of iPSC clones and analysed the data. R.D. performed teratoma experiments. X.X. and D.V.K.P. wrote the manuscript. U.Z. and W.E. edited the manuscript.

## Additional information

**How to cite this article:** Xu, X. *et al*. *Dppa3* expression is critical for generation of fully reprogrammed iPS cells and maintenance of Dlk1-Dio3 imprinting. *Nat. Commun.* 6:6008 doi: 10.1038/ncomms7008 (2015).

## Supplementary Material

Supplementary Information Supplementary Figures 1-7 and Supplementary Tables 1-2
