# Supplementary material for: Dppa3 expression is critical for generation of fully reprogrammed iPS cells and maintenance of Dlk1-Dio3 imprinting
Source: Nat Commun. 2015 Jan 23;6:6008. doi: 10.1038/ncomms7008 (PMC4354275; doi:10.1038/ncomms7008)
Supplement: Supplementary Information — Supplementary Figures 1-7 and Supplementary Tables 1-2 [file ncomms7008-s1.pdf]

Supplementary figure 1

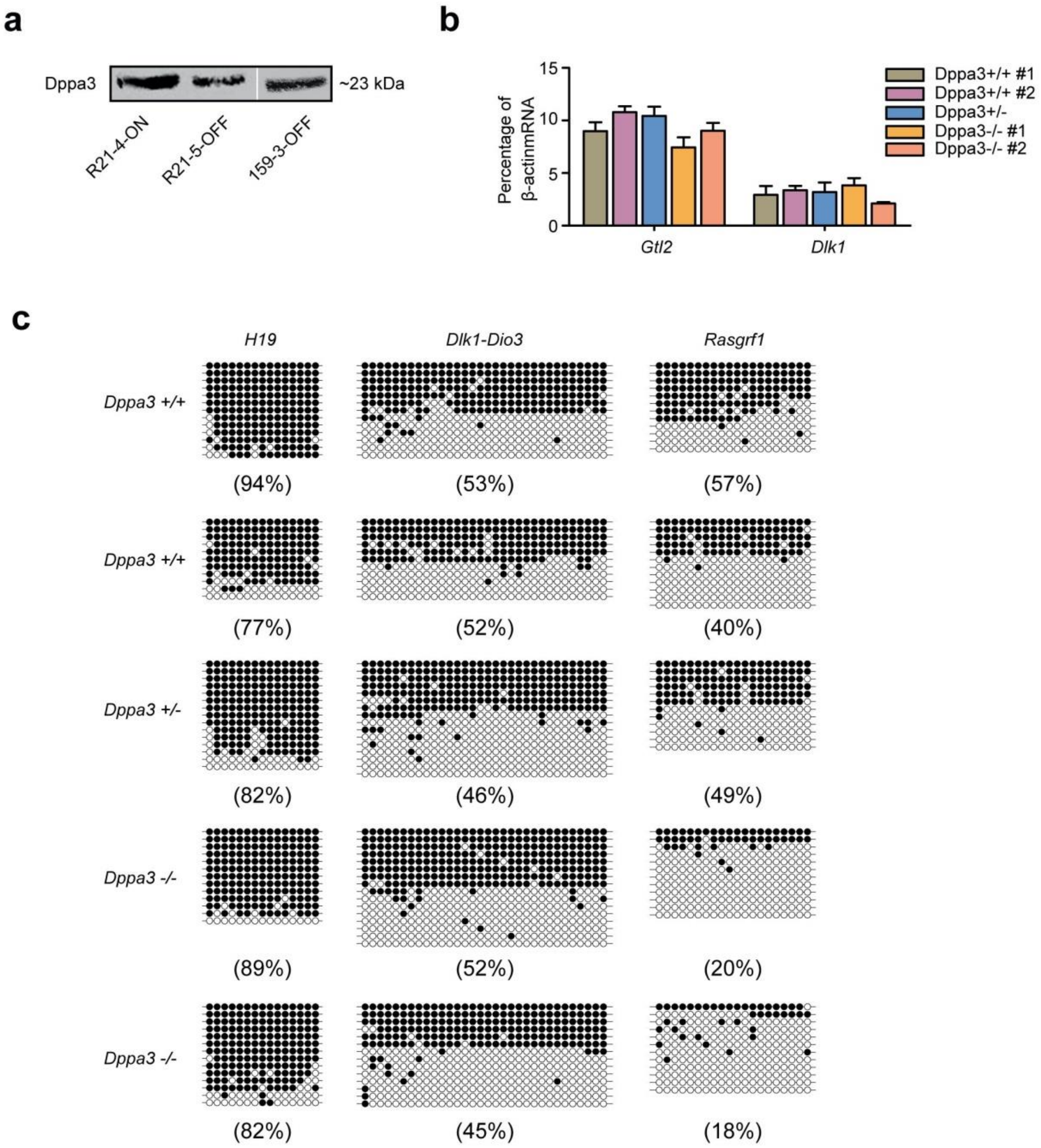

Supplementary Figure 1. Analysis of Dppa3 expression and *Dlk1-Dio3* imprinting status in previously reported iPSCs and ESCs. (a) Western blot analysis showing Dppa3 expression in previously generated iPSCs[6] (*Gtl2*^{on}: R21-4-ON and *Gtl2*^{off}: R21-5-OFF, 159-3-OFF). **(b)** qRT-PCR data showing the expression of *Gtl2* and *Dlk1* in wild-type (*Dppa3*$^{+/+}$), heterozygous (*Dppa3*$^{+/-}$), and homozygous (*Dppa3*$^{-/-}$) ESCs. Gene expression and associated error bars, representing mean ±s.d. (n=3), were normalized to housekeeping gene, *β-actin*, and presented as percentage of expression. **(c)** DNA methylation analysis of paternally imprinted *H19*, *Dlk1-Dio3*, and *Rasgrf1* loci in wild-type (*Dppa3*$^{+/+}$), heterozygous (*Dppa3*$^{+/-}$), and homozygous (*Dppa3*$^{-/-}$) ESCs.

Supplementary figure 2

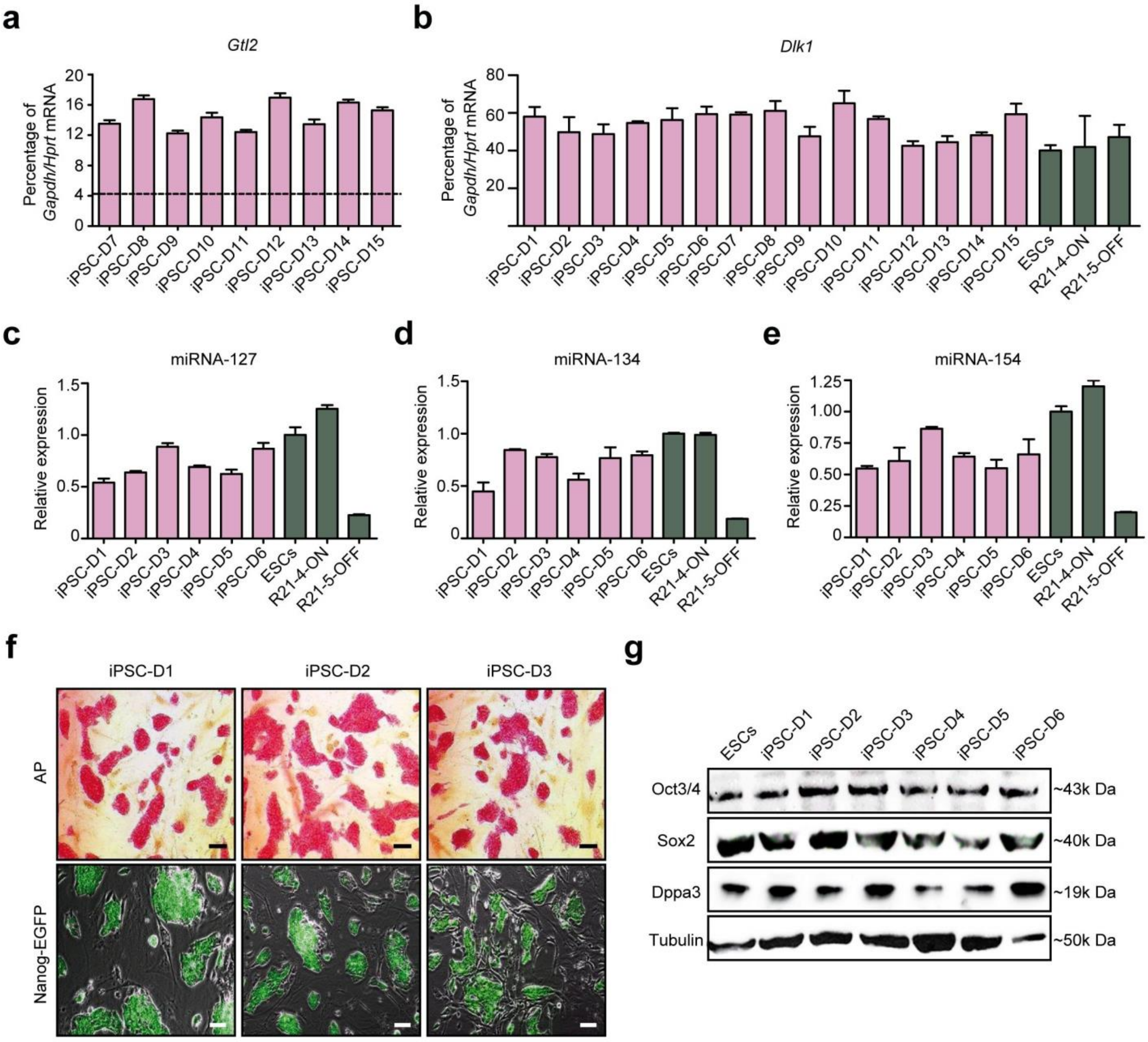

Supplementary Figure 2. Expression of genes and miRNAs encoded by the *Dlk1-Dio3* cluster and pluripotency characteristics of iPSCs generated using *Dppa3*. (a) qRT-PCR data showing the expression of *Gtl2* in iPSC clones generated using OSKM+D. *Gtl2* RNA expression and associated error bars, representing mean ±s.d. (*n*=3), were normalized to housekeeping genes, *Gapdh* and *Hprt*, and presented as percentage of expression. **(b)** qRT-PCR data showing the expression of *Dlk1* in iPSC clones generated using OSKM+D. *Dlk1* RNA expression and associated error bars, representing mean ±s.d. (*n*=3), were normalized to housekeeping genes, *Gapdh* and *Hprt*, and presented as percentage of expression. *Dlk1* expression in ESCs, R21-4-ON (*Gtl2*^{on}), and R21-5-OFF (*Gtl2*^{off}) was used as controls. **(c–e)** qRT-PCR data showing expression of miRNA-127 **(c),** miRNA-134 **(d),** and miRNA-154 **(e)** in OSKM+D iPSCs (purple). cDNA derived from ESCs, R21-4-ON (*Gtl2*^{on}), and R21-5-OFF (*Gtl2*^{off}) was used as controls. *miRNA* expression and associated error bars, representing mean ±s.d. (*n*=3), were normalized to expression level in ESCs. **(f)** Cytochemical staining demonstrates AP activity (upper panels) and fluorescence images show activation of the pluripotency reporter *Nanog*-EGFP (lower panels) in indicated iPSC colonies generated using OSKM+D. Scale bars correspond to 200 µm. **(g)** Western blots showing the expression of pluripotency marker genes Oct3/4, Sox2, and Dppa3 in indicated OSKM+D iPSCs. Total protein extracts from ESCs was used as a control. All blots were reprobed with an anti-tubulin antibody, and one representative blot shows loading control.

Supplementary figure 3

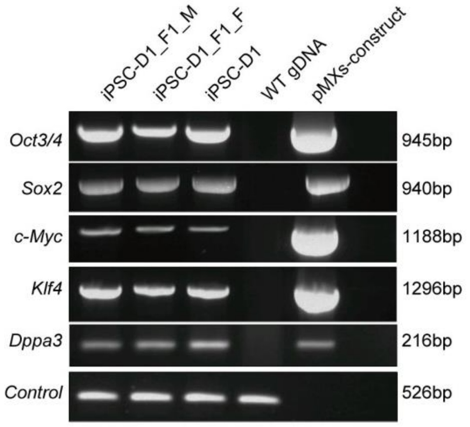

Supplementary Figure 3. Confirmation of germline transmission of iPSC generated using Dppa3. Genotyping PCR showing the presence of OSKM+D constructs in two F1 offspring (iPS-D1_F1_M and iPS-D1_F1_F) derived from the iPS-D1 chimera. Genomic DNA from iPS-D1 and wild-type ESCs (WT gDNA) served as positive and negative controls, respectively. Similarly, plasmid DNAs of OSKMD constructs (pMXs-construct) and water served as positive and negative controls, respectively. The sizes of the PCR fragments are indicated on the right.

Supplementary figure 4

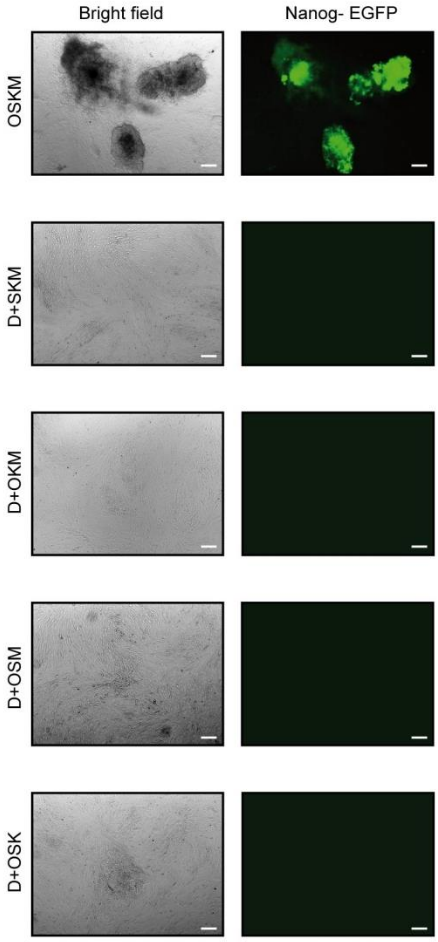

Supplementary Figure 4. Dppa3 cannot substitute any Yamanaka's factors to generate iPSCs. Bright-field images (left panel) showing the cellular morphology and fluorescence images (right panel) showing *Nanog*-EGFP fluorescent signal in *Nanog*-EGFP fibroblasts undergoing reprogramming with OSKM (*Oct3/4*; *Sox2*; *Klf4*; *c-Myc*) alone, *Dppa3* (D) replacing *Oct3/4* (D+SKM), *Dppa3* replacing *Sox2* (D+OKM), *Dppa3* replacing *Klf4* (D+OSM), and *Dppa3* replacing *c-Myc* (D+OSK) on day 21 of reprogramming.

Supplementary figure 5

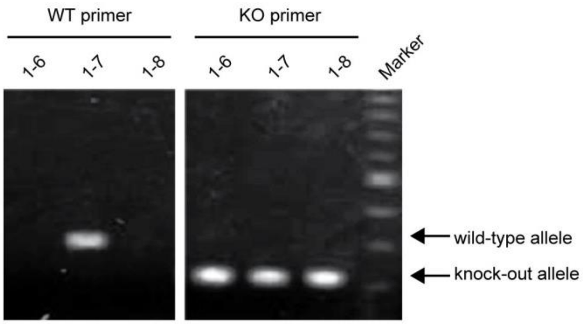

No.	Sex	Genotype	Oct4-EGFP
1-6	♀	KO	+
1-7	♂	Het	+
1-8	♂	KO	+

Supplementary Figure 5. Confirmation of *Dppa3*-KO/*Oct3/4*-EGFP genotype in fibroblasts. (Upper panel) Agarose gel electrophoresis of genotyping PCR products amplified from heterozygous (1-7) and homozygous (1-6 and 1-8) *Dppa3*-KO fibroblasts. The primers (WT or KO) are indicated on top. (Lower panel) Table shows sex of clones and results of genotyping.

Supplementary figure 6

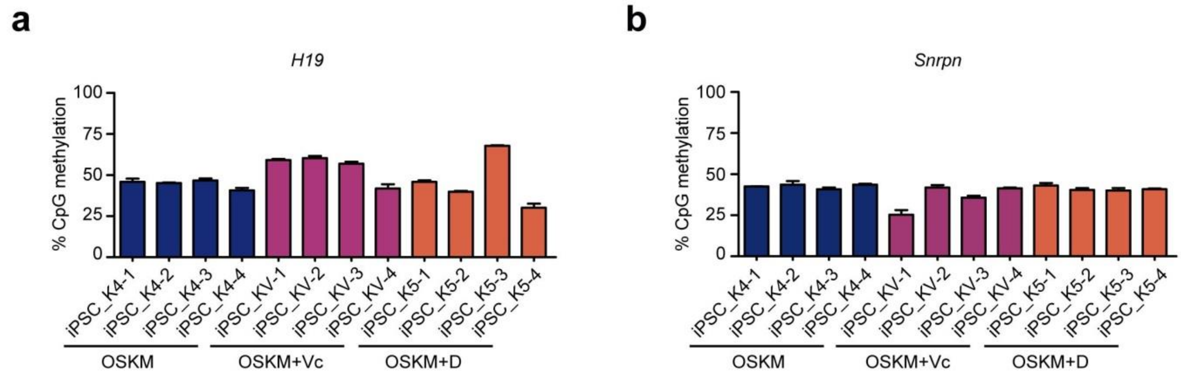

Supplementary Figure 6. *H19* and *Snrpn* imprinting status in *Dppa3*-KO-derived iPSCs. DNA methylation analysis of the *H19* **(a)** and *Snrpn* **(b)** imprinting regions in iPSCs generated from *Dppa3*-KO fibroblasts with OSKM-only (blue), OSKM+Vc (purple), and OSKM+Dppa3 (red). Error bars represent mean ±s.d. (*n*=2).

Supplementary figure 7

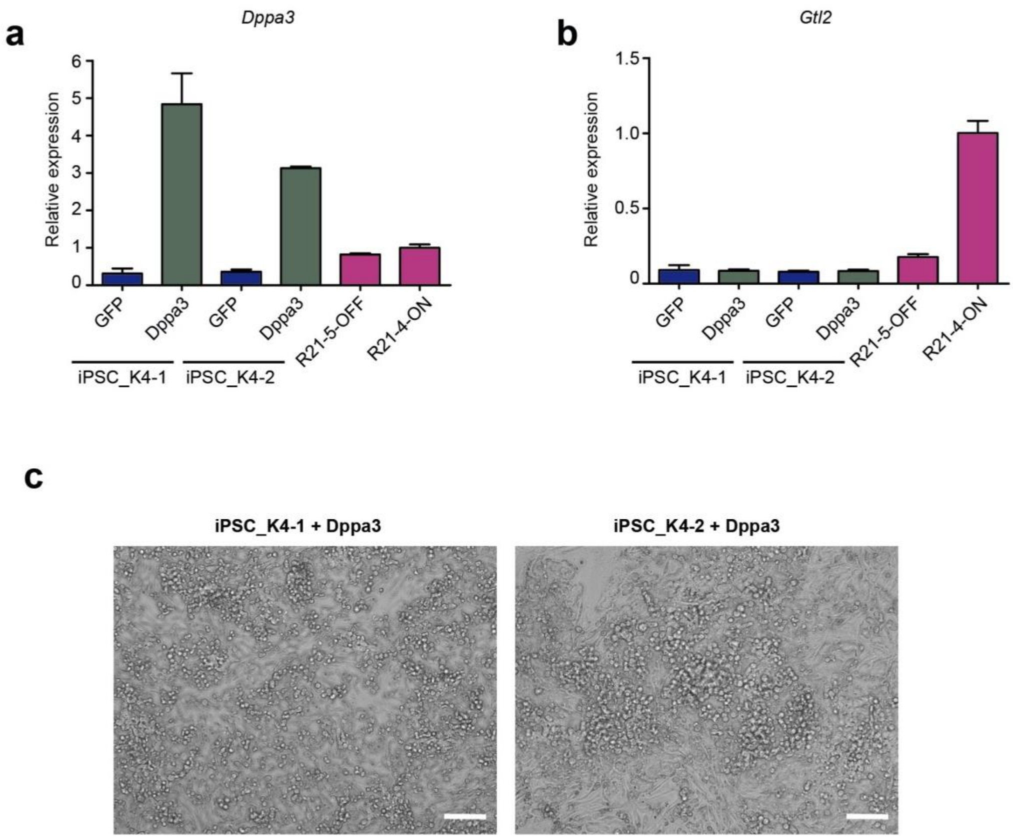

Supplementary Figure 7. Exogenous Dppa3 can not rescue pre-iPSC clones. qRT-PCR data showing the expression of *Dppa3* **(a)** and *Gtl2* **(b)** in *Dppa3*-KO MEFs-derived pre-iPSC clones (iPSC_K4-1 and iPSC_K4-2) that were transduced with either *GFP* or *Dppa3* retroviral vectors and cultured for 2-weeks. Gene expression and associated error bars, representing mean ±s.d. (*n*=2), were normalized to expression level in *Gtl2*^{on} iPSC clone[6] (R21-4-ON). **(c)** Bright-field images showing no change in colony morphology after the addition of exogenous *Dppa3*. Scale bars correspond to 200 μm.

Supplementary Table 1. Antibodies used in this study

Antibody	Source	Dilutions used
Oct3/4	ab19857 (Abcam)	1:500
Oct3/4	MAB4419 (Millipore)	1:100
Sox2	ab59776 (Abcam)	1:1,000
α-Tubulin	Sig T5168 (Sigma)	1:5,000
Dppa3	ab19878 (Abcam)	1:1,000
H3K4me3	39159 (Active Motif)	1:1000
H3K27me3	07-449 (Millipore)	1:1000
SSEA1	ab16285 (Abcam)	1:100
H3	ab1791 (Abcam)	1:2,500

Supplementary Table 2. Primers used in this study

Gene	FORWARD PRIMER SEQUENCE	REVERSE PRIMER SEQUENCE
**qRT-PCR Primers**		
Gtl2	TTGCACATTTCCTGTGGGAC	AAGCACCATGAGCCACTAGG
Dlk1	CCC AGG TGA GCT TCG AGT G	GGA GAG GGG TAC TCT TGT TGA G
Dppa3	CGGGGTTTAGGGTTAGCTTT	GGACCCTGAAACTCCTCAGA
Oct3/4	GGAAAGCAACTCAGAGGGAAC	GTGTACCCCAAGGTGATCCTC
Nanog	TTACAAGGGTCTGCTACTGAGTG	CAGGACTTGAGAGCTTTTGTTTG
Rex1	GGGTCCAAGGAGCTGAACTCC	CAGCACAGTGAGGCGATCCTG
Dnmt3l	CTTCTAGCCGATTACATCAATGG	AGTACCACATCCACACTGTCCTC
Essrb	CATGAAATGCCTCAAAGTGGG	AAATCGGCAGGTTCAGGTAG
Dppa5	GTGGGTGAAAGTTCCTGAAGAC	GAGATTCCAGGTTCTTCAGCTC
Gapdh	TCGTCCCGTAGACAAAATGG	TTGAGGTCAATGAAGGGGTC
Hprt	AGCCCCAAAATGGTTAAGGTTGC	TTGCAGATTCAACTTGCGCTCAT
**qRT-PCR primers used to test the endogenous gene expression**		
Nanog	CCAGGTTCCTTCCTTCTTCC	GGTGAGATGGCTCAGTGGAT
Oct3/4	TAGGTGAGCCGTCTTTCCAC	GCTTAGCCAGGTTCGAGGAT’
Sox2	CTGGACTGCGAACTGGAGAA	CTAGTCGGCATCACGGTTTT
**Retroviral vector primers**		
Dppa3	GAATTCGATGGAGGAACCATCAGAGAAA	GAATTCATTCTTCCCGATTTTCGCATT
IAP gag	AGCAGGTGAAGCCACTG	CTTGCCACACTTAGAGC
SINE B1	GTGGCGCACGCCTTTAATC	GACAGGGTTTCTCTGTGTAG
LINE L1	TTTGGGACACAATGAAAGCA	CTGCCGTCTACTCCTCTTGG
**ChIP primers**		
Actinb	CCCCAACACACCTAGCAAAT	ACTGCCCCATTCAATGTCTC
Dppa3_p1	GATTGCGCAGGGTTAGTTTT	GGCTTCACCTGAGCTACACC
Dppa3_p2	CCTAAAGGTGTAGCTCAGGTGAAG	GGGGGTCTGGTTTCCTTTTA
Gtl2_IGDMR_P1	TTTACCGTGGCACAGATTCA	CTGTGAGGCACTTGGCCTAT

Gtl2_IGDMR_P2	CACAGCAAAAGTGCATGGAT	CCATGGCACAACTACACAGG
Gtl2_IGDMR_P3	TCCCATACCAAGCACAATGA	GTCCACAGCACATCTCCGTA
Gtl2_IGDMR_P4	GCTTTGGAATTCCTGATGGA	GGCTACAGCAGGGAGACAAG
Gtl2_IGDMR_P5	AACAGTGTCTTCCCCCACTG	TCCCAGAGTTTCTCGGTCTT
Gtl2_IGDMR_P6	CTTTTGTTGACCACAACCCTTG	AATCCCACCACAGCTTCTTAGC
Gtl2_IGDMR_P7	GCACTGAGGCACAATTCAGA	GCTTAGGGGTTTCTCCAACC
Gtl2_IGDMR_P8	CCACAAAAACCTCCCTTTCA	GGCTTCCTTGTTCTGACTGC
**Virus integration verification primers**		
Oct4_R_geno	CAGTGTGGTGGTACGGGAAATCAC	CTGGGAAAGGTGTCCCTGTA
Sox_R_geno	CAGTGTGGTGGTACGGGAAATCAC	TGGGCCATGTGCAGTCTAC
Klf4_R_geno	CAGTGTGGTGGTACGGGAAATCAC	CTGTGTGAGTTCGCAGGTGT
cMyc_R_geno	CAGTGTGGTGGTACGGGAAATCAC	TCTGACGTTCCAAGACGTTG
Dppa3_R_geno	CAGTGTGGTGGTACGGGAAATCAC	ACACCGGGGTTTAGGGTTAG
**miRNA expression analysis primers**		
miR-127	TCGGATCCGTCTGAGCTTG	
miR-134	TGTGACTGGTTGACCAGAGGG	
miR-154	TAGGTTATCCGTGTTGCCTTCG	
miR-329	CAACACACCCAGCTAACCTTTTT	
miR-342	CACACAGAAATCGCACCCG	
U6	TGGCCCCTGCGCAAGGATG	
***Dlk1-Dio3* IG-DMR methylation analysis primers**		
PCR primers	GTGGTTTGTTATGGGTAAGTTT	Biotinylated-CCCTTCCCTCACTCCAAAAAT TAA
Sequencing	GTTTTATGGTTTATTGTATATAATG	
